# Range-doppler algorithm of multireceiver synthetic aperture sonar using nonuniform signal

**DOI:** 10.1371/journal.pone.0343983

**Published:** 2026-03-24

**Authors:** Pan Huang, Yan Fan, Xiaojie Teng, Jingjing Tan, Lijie Guo

**Affiliations:** 1 School of Mathematics and Statistics, Weifang University, Weifang, Shandong, China; 2 College of Ecological Engineering, Shandong Ecology and Environment University, Weifang, Shandong, China; 3 Basic Teaching Department, Shandong Vocational College of Science and Technology, Weifang, Shandong, China; Northwest Normal University, CHINA

## Abstract

When the structures of multireceiver synthetic aperture sonar (SAS) are applied, imaging techniques are not always able to acquire images with outstanding resolution in nonuniform situations. In order to cope with this issue, we present a robust imaging algorithm in this paper. Based on our method, the receivers adopted by imaging algorithms are firstly determined. Then, we model the multireceiver SAS spectrum made up of the monostatic one and bistatic one. The bistatic portion is resolved via the application of sub-block filtering in the spectral domain. In the range-Doppler domain, the spectrums of determined receivers are coherently added. In the spectral domain, the compression in the range direction relying on multiplications is handled. With the help of interpolations, the coupling between both directions is removed in the range-Doppler domain, where the subsequent step is to do the compression in the azimuth direction. Our method can well focus the SAS datasets with the slight nonuniform case and redundant receiver case. However, traditional method just works well with the slight nonuniform case. In comparison with traditional method, our method can get high-resolution image all the time. The results based on simulations and experiments fully validate our method.

## 1. Introduction

As a mature underwater imaging technology [1,2], synthetic aperture sonar (SAS) [3–5] can carry out the imaging of the seafloor, and it is very important for the ocean observation [6–8] and underwater imagery. With the increasing demand of underwater imagery [9–11], SAS is viewed to be the good choice of small target detection [12–15] and recognition [16–20]. To achieve the large-scale swath, the emitter should emit signal with low pulse repetition frequency (PRF). To prevent spectrum ambiguity and retain outstanding resolution in the azimuth direction, it is necessary to apply large PRF which must be much broader than the Doppler bandwidth. As a result, the requirement for outstanding resolution and large-scale imaging area cannot be met by relying on traditional monostatic SAS systems. In order to cope with this contradiction, multireceiver synthetic aperture sonar (SAS) [21–24] indicating an emitter and a receiver array are mounted on the same sonar carrier is proposed. Using multiple receivers in the azimuth dimension, echoed signal from multiple receivers can be obtained at the same time. The demand of instantaneous sampling in range is alleviated by the enhancement of spatial sampling in azimuth. Compared to monostatic SAS, it simultaneously owns the ability of large-scale swath and high resolution [25,26].

For multireceiver SAS, the imagery is a core job. Based on the range-Doppler (RD) algorithm [27–29], the SAS imagery can be decomposed into three main steps, i.e., range compression, decoupling and azimuth compression. Since RD algorithm is simple and easily understood, it is popularly used by various systems. In [30], the RD algorithm based on hyperbolic slant range is presented. However, the higher order error which would have an impact on the imaging quality is ignored by this technique. The 2D spectrum and RD algorithm are designed in [31] via applying the stationary phase approach and the expansion of Taylor series with respect to the SAS overall range. In general, this method owns many approximations resulting in residual phase error. In [32], Zhongs et al develop the RD algorithm based on the lower-order Taylor series expansion of SAS overall range. With this method, the influence of approximation error on the migration curve is not corrected, and the imaging performance would be introduced by the higher sidelobes. In [33], Yangs et al present the RD algorithm based on the numerical computation technique, which is utilized to acquire stationary point and range migration function. In practice, the range migration on the basis of the calculated stationary point disregards the Doppler impacts giving rise to further phase error.

In general, the above methods need that the SAS system works in the ideal case [34,35], where the sonar moves precisely the half aperture of receiver array. Usually, we call it the uniform sampling case. Unluckily, the ocean is disturbed by the wind, current, etc. The moving speed of SAS system cannot be always met the constant requirement [36–38]. Therefore, the strict requirement cannot be satisfied, and the nonuniform sampling data would be caused in the azimuth dimension. When the SAS system suffers from nonuniform sampling, the above imaging methods cannot generate the high-resolution results. In order to cope with this issue, nonuniform data should be employed to recover the uniform data. In [39], the spectrum of nonuniform data is considered to be the weighted spectrum of uniform signal. Additionally, the weighting function [40] is barely affected by the azimuth and range frequencies. The main work of this method focuses on the spectrum calculation of nonuniform data and the weighting function. However, both works are time-consuming. Based on the filter bank (FB) method [41,42], each receiver data is considered to be the filtering results of nonuniform signal, and each receiver signal is uniformly sampled. From the filter inversion concept, the uniform signal can be efficiently reconstructed based on each receiver spectrum and inversion of filter bank. Some researchers have demonstrated that both methods are equivalent [43]. To some degree, both methods relax the SAS moving requirement between two adjacent pulses compared to traditional methods. However, this relaxation is just limited to a single receiver aperture in the azimuth dimension. When the deviation from the uniform sampling case is more than a receiver aperture in the azimuth dimension, the overlapped receivers and redundant signal between two adjacent pulses are caused. When the SAS system works in this case, neither of two uniform signal reconstruction techniques could provide images with excellent resolution.

Regarding our work of the paper, an imagery method which can simultaneously process the SAS data in the uniform and nonuniform cases is offered. With our method, we firstly choose the receivers adopted by our imaging algorithm. Based on this, we model the multireceiver SAS spectrum consisting of monostatic one and bistatic one, which is compensated for each receiver data in the spectrum domain. In the Doppler domain, the spectrums associated with chosen receivers are coherently superposed. We carry out the compression in the spectral domain of range dimension. The data is then converted into the range-time and azimuth-Doppler domain, the range migration is hereafter removed by correcting the decoupling between both dimensions with interpolations. After this operation, the compression and correction of shifting in azimuth is carried out. Compared to traditional method, our solution can greatly enhance the imaging capability.

The construction of our work is presented below. Section 2 discloses the geometric configuration and model of SAS system. Hereafter, we discuss our method in detail in the next section. The suggested approach is validated in section 4 with the help of tests and simulations. Lastly, the work in this paper is concluded.

## 2. Geometric configuration and model

**Fig 1:**
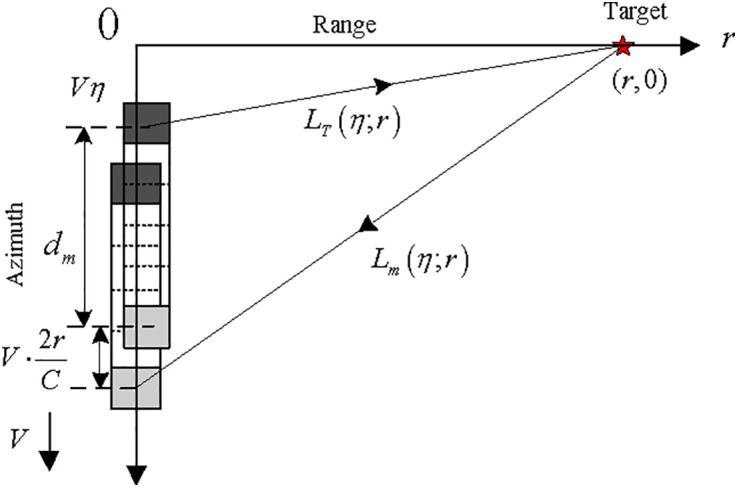
Geometric configuration of SAS system.


Fig 1 illustrates the geometry of multireceiver SAS system consisting of one transmitter and *M* equally distributed receiver sensors. The transmitter is filled with black while no colors are filled by the receivers. The system [44] moves in the azimuth dimension with an ideal velocity *V*. We suppose that the position of transmitter in the azimuth dimension is 0 when the system plans to work. After some working time η, the azimuth position of transmitter is Vη. The range history from transmitter location to an assumed object (*r*, 0) can be represented as

$$L_{T}\left(\eta ;r\right)=\sqrt{r^{\text{2}}+V^{\text{2}}\eta^{\text{2}}}$$ (1)

In Eq. (1), the slow time and range are denoted by η and *r*, individually. The receiver location is $V\eta +d_{m}$ in the azimuth dimension, and $d_{m}$ denotes the limited distance of the subsystem made up of the sender and the *m*th receiver sensors. The distance from this receiver location to the assumed object (*r*, 0) is expressed by


$$L_{m}\left(\eta ;r\right)=\sqrt{r^{\text{2}}+{\left(V\eta +2V\frac{r}{C}+d_{m}\right)}^{\text{2}}}$$ (2)

We assume that the chirp waveform [45,46] is emitted, and the echo collected by the *m*th receiver is provided by


$$s_{m}\left(\varsigma ,\eta \right)=s\left(\varsigma -\frac{L_{T}\left(\eta ;r\right)+L_{m}\left(\eta ;r\right)}{C}\right)\cdot exp\left\{-\text{j}2\pi \nu_{c}\frac{L_{T}\left(\eta ;r\right)+L_{m}\left(\eta ;r\right)}{C}\right\}$$ (3)

where C and $\nu_{c}$ represent the acoustic velocity and carrier frequency, individually. The fast time is denoted by the symbol ς.

## 3. Multireceiver SAS RD algorithm

### 3.1. 2D Spectrum

The main job of this section is to deduce the 2D spectrum of echo shown in Eq. (3).

Before discussing our imaging algorithm, we firstly present 2D spectrum [47–49], which is an indispensable work of fast focusing algorithms. Depending on the Fourier transform (FT) with respect to the fast time, Eq. (3) is converted into the spectral domain in the range direction and the result is written as


$$S_{m}\left(\nu_{\varsigma },\eta \right)=S\left(\nu_{\varsigma }\right)\cdot exp\left\{-\text{j}2\pi \left(\nu_{c}+\nu_{\varsigma }\right)\frac{L_{T}\left(\eta ;r\right)+L_{m}\left(\eta ;r\right)}{C}\right\}$$ (4)

where the instantaneous frequency related to the fast time is shown by $\nu_{\varsigma }$. $S\left(\nu_{\varsigma }\right)$ is the spectrum of s(ς) in Eq. (3).

In azimuth direction, exploiting the FT generates the 2D spectrum, and it is indicated by


$$\begin{array}{c} {\tilde{S}}_{m}\left(\nu_{\varsigma },\nu_{\eta }\right)=S\left(\nu_{\varsigma }\right)\cdot \int exp\left\{-\text{j}2\pi \left(\nu_{c}+\nu_{\varsigma }\right)\frac{L_{T}\left(\eta ;r\right)+L_{m}\left(\eta ;r\right)}{C}-\text{j}2\pi \nu_{\eta }\eta \right\}d\eta \mathrm{\backslash hfill}\\ =S\left(\nu_{\varsigma }\right)\cdot \int exp\left\{-\text{j}2\pi \left(\nu_{c}+\nu_{\varsigma }\right)\frac{L_{T}\left(\eta ;r\right)}{C}-\text{j}\pi \nu_{\eta }\eta --\text{j}2\pi \left(\nu_{c}+\nu_{\varsigma }\right)\frac{L_{m}\left(\eta ;r\right)}{C}-\text{j}\pi \nu_{\eta }\eta \right\}d\eta \mathrm{\backslash hfill} \end{array}$$ (5)

where the Doppler frequency is shown by $\nu_{\eta }$ in the azimuth dimension.

For ease of clarification, we define the emitter phase and receiver phase $\delta_{T}\left(\nu_{\varsigma },\eta \right)=\text{2}\pi \frac{\nu_{c}+\nu_{\varsigma }}{C}L_{T}(\eta ;r)+\pi \nu_{\eta }\eta$ and $\delta_{m}\left(\nu_{\varsigma },\eta \right)=\text{2}\pi \frac{\nu_{c}+\nu_{\varsigma }}{C}L_{m}(\eta ;r)+\pi \nu_{\eta }\eta$. Based on the stationary phase principle [50], two stationary phase points corresponding to emitter phase and receiver phase are developed, and they are indicated by ${\overset{⌣}{\eta }}_{T}$ and ${\overset{⌣}{\eta }}_{m}$, respectively. With the quadratic expansion, the emitter and receiver phases are given by


$$\delta_{T}\left(\nu_{\varsigma },\eta \right)\approx \delta_{T}\left(\nu_{\varsigma },{\overset{\mathrm{\backslash lower}0.5em\text{\textbackslash{}smash\{\textbackslash{}scriptscriptstyle\textbackslash{}smile}}{\}}\eta }_{T}\right)+\delta_{T}^{\text{'}}\left(\nu_{\varsigma },{\overset{\mathrm{\backslash lower}0.5em\text{\textbackslash{}smash\{\textbackslash{}scriptscriptstyle\textbackslash{}smile}}{\}}\eta }_{T}\right)\left(\eta -{\overset{\mathrm{\backslash lower}0.5em\text{\textbackslash{}smash\{\textbackslash{}scriptscriptstyle\textbackslash{}smile}}{\}}\eta }_{T}\right)+0.5\delta_{T}^{\text{''}}\left(\nu_{\varsigma },{\overset{\mathrm{\backslash lower}0.5em\text{\textbackslash{}smash\{\textbackslash{}scriptscriptstyle\textbackslash{}smile}}{\}}\eta }_{T}\right){\left(\eta -{\overset{\mathrm{\backslash lower}0.5em\text{\textbackslash{}smash\{\textbackslash{}scriptscriptstyle\textbackslash{}smile}}{\}}\eta }_{T}\right)}^{\text{2}}$$ (6)

$$\delta_{m}\left(\nu_{\varsigma },\eta \right)\approx \delta_{m}\left(\nu_{\varsigma },{\overset{\mathrm{\backslash lower}0.5em\text{\textbackslash{}smash\{\textbackslash{}scriptscriptstyle\textbackslash{}smile}}{\}}\eta }_{m}\right)+\delta_{m}^{\text{'}}\left(\nu_{\varsigma },{\overset{\mathrm{\backslash lower}0.5em\text{\textbackslash{}smash\{\textbackslash{}scriptscriptstyle\textbackslash{}smile}}{\}}\eta }_{m}\right)\left(\eta -{\overset{\mathrm{\backslash lower}0.5em\text{\textbackslash{}smash\{\textbackslash{}scriptscriptstyle\textbackslash{}smile}}{\}}\eta }_{m}\right)+0.5\delta_{m}^{\text{''}}\left(\nu_{\varsigma },{\overset{\mathrm{\backslash lower}0.5em\text{\textbackslash{}smash\{\textbackslash{}scriptscriptstyle\textbackslash{}smile}}{\}}\eta }_{m}\right){\left(\eta -{\overset{\mathrm{\backslash lower}0.5em\text{\textbackslash{}smash\{\textbackslash{}scriptscriptstyle\textbackslash{}smile}}{\}}\eta }_{m}\right)}^{\text{2}}$$ (7)

where $\delta_{T}^{\text{'}}\left(\nu_{\varsigma },{\overset{\mathrm{\backslash lower}0.5em\text{\textbackslash{}smash\{\textbackslash{}scriptscriptstyle\textbackslash{}smile}}{\}}\eta }_{T}\right)$ and $\delta_{m}^{\text{'}}\left(\nu_{\varsigma },{\overset{\mathrm{\backslash lower}0.5em\text{\textbackslash{}smash\{\textbackslash{}scriptscriptstyle\textbackslash{}smile}}{\}}\eta }_{m}\right)$ represent the first-order derivative with respect to the slow time. The first-order derivative is zero according to the stationary phase principle, i.e., $\delta_{T}^{\text{'}}\left(\nu_{\varsigma },{\overset{\mathrm{\backslash lower}0.5em\text{\textbackslash{}smash\{\textbackslash{}scriptscriptstyle\textbackslash{}smile}}{\}}\eta }_{T}\right)=0$ and $\delta_{m}^{\text{'}}\left(\nu_{\varsigma },{\overset{\mathrm{\backslash lower}0.5em\text{\textbackslash{}smash\{\textbackslash{}scriptscriptstyle\textbackslash{}smile}}{\}}\eta }_{m}\right)=0$. $\delta_{T}^{\text{''}}\left(\nu_{\varsigma },{\overset{\mathrm{\backslash lower}0.5em\text{\textbackslash{}smash\{\textbackslash{}scriptscriptstyle\textbackslash{}smile}}{\}}\eta }_{T}\right)$ and $\delta_{m}^{\text{''}}\left(\nu_{\varsigma },{\overset{\mathrm{\backslash lower}0.5em\text{\textbackslash{}smash\{\textbackslash{}scriptscriptstyle\textbackslash{}smile}}{\}}\eta }_{m}\right)$ are the second-order derivative.

Based on Eq. (6) and Eq. (7), the emitter and receiver phases in Eq. (5) are replaced by the approximated terms, and the resultant integral shown in Eq. (5) is reformulated to


$$\begin{array}{cc} {\tilde{S}}_{m}\left(\nu_{\varsigma },\nu_{\eta }\right)= & \;S\left(\nu_{\varsigma }\right)exp\left\{-\text{j}\left[\delta_{T}\left(\nu_{\varsigma },{\overset{\mathrm{\backslash lower}0.5em\text{\textbackslash{}smash\{\textbackslash{}scriptscriptstyle\textbackslash{}smile}}{\}}\eta }_{T}\right)+\delta_{m}\left(\nu_{\varsigma },{\overset{\mathrm{\backslash lower}0.5em\text{\textbackslash{}smash\{\textbackslash{}scriptscriptstyle\textbackslash{}smile}}{\}}\eta }_{m}\right)\right]\right\}\\ & \underset{-\infty }{\int^{\infty }}exp\left\{-0.5\text{j}\left[\delta_{T}^{\text{''}}\left(\nu_{\varsigma },{\overset{\mathrm{\backslash lower}0.5em\text{\textbackslash{}smash\{\textbackslash{}scriptscriptstyle\textbackslash{}smile}}{\}}\eta }_{T}\right){\left(\eta -{\overset{\mathrm{\backslash lower}0.5em\text{\textbackslash{}smash\{\textbackslash{}scriptscriptstyle\textbackslash{}smile}}{\}}\eta }_{T}\right)}^{\text{2}}+\delta_{m}^{\text{''}}\left(\nu_{\varsigma },{\overset{\mathrm{\backslash lower}0.5em\text{\textbackslash{}smash\{\textbackslash{}scriptscriptstyle\textbackslash{}smile}}{\}}\eta }_{m}\right){\left(\eta -{\overset{\mathrm{\backslash lower}0.5em\text{\textbackslash{}smash\{\textbackslash{}scriptscriptstyle\textbackslash{}smile}}{\}}\eta }_{m}\right)}^{\text{2}}\right]\right\}d\eta \end{array}$$ (8)

Exploiting the stationary phase principle again, Eq. (8) would be solved and the analytical expression of 2D spectrum is provided by


$$\begin{array}{cc} {\tilde{S}}_{m}\left(\nu_{\varsigma },\nu_{\eta }\right)= & S\left(\nu_{\varsigma }\right)exp\left\{-\text{j}\frac{\pi {\left(d_{m}+2\frac{r}{C}V\right)}^{\text{2}}{\left[{\left(\nu_{\text{c}}+\nu_{\varsigma }\right)}^{\text{2}}-\nu_{\eta }^{\text{2}}\frac{C^{\text{2}}}{4V^{\text{2}}}\right]}^{3/2}}{2{\left(\nu_{\text{c}}+\nu_{\varsigma }\right)}^{\text{2}}Cr}\right\}exp\\ & \left\{\text{j}\pi \nu_{\eta }\frac{d_{m}}{V}\right\}exp\left\{-\text{j}\frac{4\pi }{C}r\sqrt{{\left(\nu_{\text{c}}+\nu_{\varsigma }\right)}^{\text{2}}-\nu_{\eta }^{\text{2}}\frac{C^{\text{2}}}{4V^{\text{2}}}}\right\}exp\left\{\text{j}2\pi \nu_{\eta }\frac{r}{C}\right\} \end{array}$$ (9)

Examining Eq. (9), the initial component represents the chirp spectrum. The subsequent two items are the bistatic phase arising from the displaced separation between emitter and receiver. The monostatic SAS spectrum is the precondition of RD algorithm, and its expression is very close to the fourth factor in(9). The stop-and-hop assumption usually applied in the radar field [51–53] results in the deviation [54], and it is shown by the final item.

**Fig 2:**
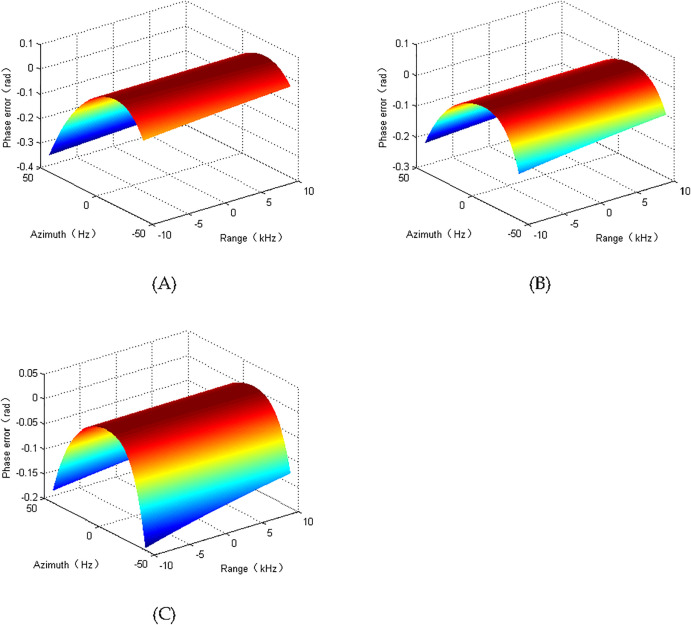
Phase error. (A) the range is 30m. (B) the range is 120m. (C) the range is 210m.

The SAS system owns 50 receivers, and the total aperture of receiver array is 1.5 m. The aperture of emitter is 0.06 m. The carrier frequency of sonar system uses 150000 Hz. 20000 Hz is used by the chirp waveform bandwidth. The interval of sent chirp is 300 ms. The sonar is operated with 2.5 m/s. The system works with uniform case. The phase error of presented spectrum shown in Eq. (9) increases with the aperture. Based on the accurate spectrum, the phase error of largest aperture is displayed in Fig 2. When the SAS focusing is performed, the error not exceeding $\frac{\pi }{\text{8}}$ would not noticeably affect the SAS quality. The maximum error of our spectrum is not exceed to $\frac{\pi }{\text{8}}$. Therefore, the preseted spectrum shown in Eq. (9) has the ability to generate high quality results.

### 3.2. Imaging processor

Based on the 2D spectrum shown in Eq. (9), the work of this part is devoted to the development of RD processor [28] for multireceiver configuration. According to the sonar moving velocity and the pulse repetition interval (PRI), the moving distance between two adjacent pulses is given by PRI×V. In practice, the azimuth sampling should be adhering to the Nyquist sampling like the time-domain sampling. The azimuth sampling is strictly carried out based on the receiver, and each receiver aperture is the sampling interval. That is to say, the moving distance per pulse should be strictly half length of the array. Since the spatial sampling interval is too small, the deviation caused by the nonuniform sampling can be neglected. This is the key of our approach. Using the real aperture of each receiver, the number of receivers exploited by the imaging processor is conformed, and it is shown as


$$N=\text{round}\left\{\frac{PRI\times V}{0.5d}\right\}$$ (10)

where *d* indicates the aperture of receiver. round{·} shows that the nearest integer is selected by the imaging processor.

Based on Eq. (9), the matched filter is used to perform the compression in range, and the filter item is written by


$$H_{MF}\left(\nu_{\varsigma }\right)=\text{conj}\left\{S\left(\nu_{\varsigma }\right)\right\}$$ (11)

where conj{·} shows the conjugate operation.

Following the aforementioned filtering, every receiver data is divided into *Q* blocks. Every block data is converted into spectral domain, and we aim to correct the second item in Eq. (9). For the *q*th block, the correction term is given by


$$H_{n,q}(\nu_{\varsigma },\nu_{\eta };r_{q})=exp\left\{\text{j}\frac{\pi {\left(d_{n}+2\frac{r_{q}}{C}V\right)}^{\text{2}}{\left[{\left(\nu_{\text{c}}+\nu_{\varsigma }\right)}^{\text{2}}-\nu_{\eta }^{\text{2}}\frac{C^{\text{2}}}{4V^{\text{2}}}\right]}^{3/2}}{2C{\left(\nu_{\text{c}}+\nu_{\varsigma }\right)}^{\text{2}}r_{q}}\right\}$$ (12)

where $r_{q}$ denotes the center of each block.

Actually, the large error after correction lies in the edge of this block. When the SAS focusing is performed, the error not exceeding $\frac{\pi }{\text{8}}$ would not noticeably affect the SAS quality. We define the block length U. Then, this relationship is shown as


$$\left|\frac{\pi {\left(d_{n}+2\frac{r_{q}}{C}V\right)}^{\text{2}}{\left[{\left(\nu_{\text{c}}+\nu_{\varsigma }\right)}^{\text{2}}-\nu_{\eta }^{\text{2}}\frac{C^{\text{2}}}{4V^{\text{2}}}\right]}^{3/2}}{2C{\left(\nu_{\text{c}}+\nu_{\varsigma }\right)}^{\text{2}}r_{q}}-\frac{\pi {\left(d_{n}+2\frac{r_{q}\pm 0.5U}{C}V\right)}^{\text{2}}{\left[{\left(\nu_{\text{c}}+\nu_{\varsigma }\right)}^{\text{2}}-\nu_{\eta }^{\text{2}}\frac{C^{\text{2}}}{4V^{\text{2}}}\right]}^{3/2}}{2C{\left(\nu_{\text{c}}+\nu_{\varsigma }\right)}^{\text{2}}\left(r_{q}\pm 0.5U\right)}\right|<\frac{\pi }{\text{8}}$$ (13)

Eq. (13) is further reformulated as


$$\left|\frac{{\left(d_{n}+2\frac{r_{q}}{C}V\right)}^{\text{2}}}{r_{q}}-\frac{{\left(d_{n}+2\frac{r_{q}\pm 0.5U}{C}V\right)}^{\text{2}}}{\left(r_{q}\pm 0.5U\right)}\right|<\frac{C{\left(\nu_{\text{c}}+\nu_{\varsigma }\right)}^{\text{2}}}{4{\left[{\left(\nu_{\text{c}}+\nu_{\varsigma }\right)}^{\text{2}}-\nu_{\eta }^{\text{2}}\frac{C^{\text{2}}}{4V^{\text{2}}}\right]}^{3/2}}$$ (14)

Based on Eq. (14), the block length is got. Consequently, the total block number *Q* is got.

All block datasets in the spectral domain are then transformed into fast time domain, where all blocks are integrated together to generate an entire receiver signal.

Subsequently, we plan to eliminate the impacts of the bistatic phase expressed by the third term in Eq. (9), and the compensation function is expressed as


$$H_{n}(\nu_{\eta };d_{n})=exp\left\{-\text{j}\pi \nu_{\eta }\frac{d_{n}}{V}\right\}$$ (15)

After this compensation, the coherent summation of all sensor spectrums is operated, and this operation is shown as


$$\tilde{S}\left(r,\nu_{\eta }\right)=\sum_{n=1}^{N}{\tilde{S}}_{n}\left(r,\nu_{\eta }\right)$$ (16)

When we complete the coherent summation, all receiver datasets are coerced into the uniform equivalent signal. To compensate the fourth term in Eq. (9), it is expanded up to the quadratic term, and the expression is shown as


$$-\frac{4\pi }{C}r\sqrt{{\left(\nu_{\text{c}}+\nu_{\varsigma }\right)}^{\text{2}}-\nu_{\eta }^{\text{2}}\frac{c^{\text{2}}}{4V^{\text{2}}}}\approx -\frac{4\pi r\beta }{\lambda }-\frac{4\pi r}{C\beta }\nu_{\varsigma }+2\pi \frac{r\lambda }{C^{\text{2}}}\left(\frac{\text{1}}{\beta^{\text{3}}}-\frac{\text{1}}{\beta }\right)\nu_{\varsigma }^{\text{2}}$$ (17)

where a temporary symbol $\beta =\sqrt{1-\frac{C^{\text{2}}\nu_{\varsigma }^{\text{2}}}{4V^{\text{2}}\nu_{\text{c}}^{\text{2}}}}$ is defined. The primary item in Eq. (17) indicates the modulation in azimuth. The subsequent item represents the range and azimuth coupling. The final factor in Eq. (17) shows the chirp characteristic resulted from the quadratic coupling of both dimensions.

In the spectral domain, the approximation error that arises from Eq. (17) is compensated. Besides, the quadratic coupling in Eq. (17) is corrected. To perform this step, the filter is supplied by


$$H(\nu_{\varsigma },\nu_{\eta };r_{s})=exp\left\{\text{j}\frac{4\pi }{C}r_{s}\sqrt{{\left(\nu_{\text{c}}+\nu_{\varsigma }\right)}^{\text{2}}-\nu_{\eta }^{\text{2}}\frac{C^{\text{2}}}{4V^{\text{2}}}}-\text{j}\frac{4\pi r_{s}\beta }{\lambda }-\text{j}\frac{4\pi r_{s}}{C\beta }\nu_{\varsigma }\right\}$$ (18)

where $r_{s}$ denotes the range of particular target in the imagery region.

Following the preceding action, the inverse Fourier transform (IFT) is applied to datasets in the range direction, and the datasets are converted into the range time domain from range frequency domain. Then, the linear coupling between both dimensions in Eq. (17) is eliminated via making use of interpolation technique, and the delay offset arising from the range cell migration is represented by


$$\Delta r=\frac{2r}{\beta }-2r$$ (19)

The term shown in Eq. (19) is not the same for each range bin. Therefore, it cannot be corrected by using the multiplex multiplication at the same time. Considering this factor, the linear coupling between both directions in Eq. (17) is cancelled via the application of interpolation technique. The physical meaning of this step eliminates the coupling between two directions.

After the former handling stage, we aim to do the matched filtering and deviation correction in azimuth. The physical meaning of this step performs the compression in the azimuth direction. We provide the handling filter shown as


$$H\left(\varsigma ,\nu_{\eta }\right)=exp\left\{\text{j}\frac{4\pi r\beta }{\lambda }\right\}exp\left\{\text{-j}2\pi \nu_{\eta }\frac{r}{C}\right\}$$ (20)

**Fig 3:**
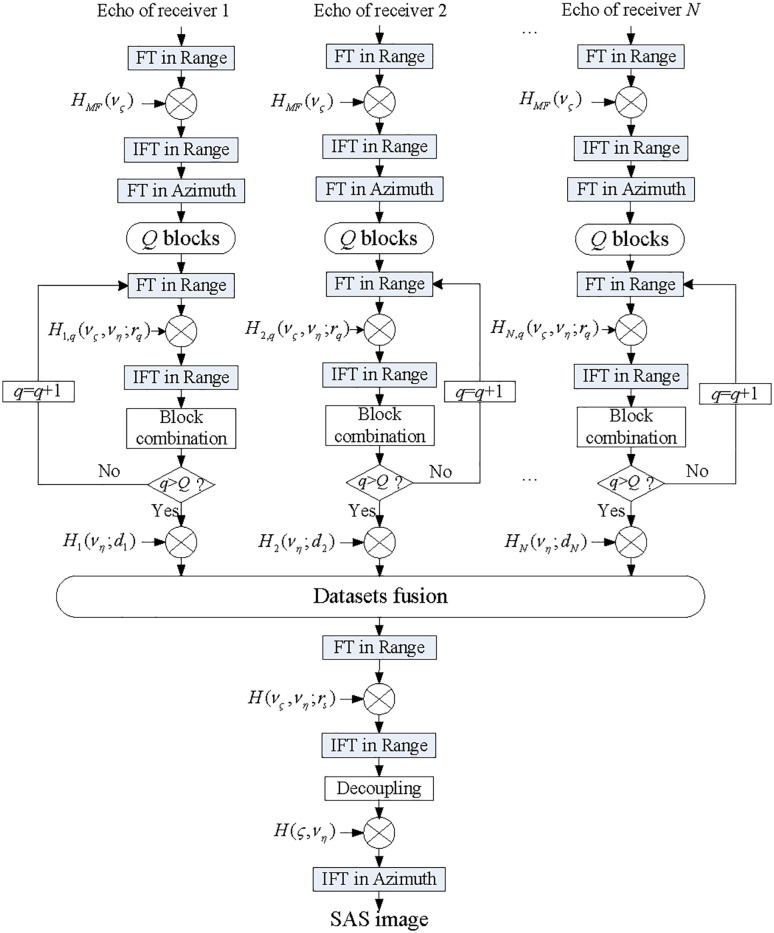
Flowchart of proposed imaging processor.

In the azimuth dimension, we do the IFT, and the image with outstanding quality is created. Based on the processing mentioned above, Fig 3 shows the processing structure of described solution.

### 3.3. Computation complexity

The basic operations used by our method mainly include the FT/IFT, multiplex multiplication and interpolation. In [55], the computation complexity for these operations has been discussed. In this section, the size of original data matrix is supposed to be $N_{r}\times N_{a}$. On the basis of this, we directly present the computation complexity of our method, and it is shown as


$$O=20N_{a}N_{r}{log}_{\text{2}}^{N_{r}}+5N_{a}N_{r}{log}_{\text{2}}^{\frac{N_{a}}{N}}+10N_{a}\frac{N_{r}}{Q}{log}_{\text{2}}^{\frac{N_{r}}{Q}}+24N_{a}N_{r}+6N_{a}\frac{N_{r}}{Q}+2\left(2M_{ker}-1\right)N_{a}N_{r}+5N_{a}N_{r}{log}_{\text{2}}^{N_{a}}$$ (21)

where *N* is the receiver numbers exploited by our imaging processor. The interpolation length is represented by $M_{ker}$.

It is apparently visible from Fig 2 that our solution mainly exploits the FT/IFT and multiplex multiplication. Therefore, the presented method has the characteristics of high efficiency.

## 4. Simulations and experiments

### 4.1. Simulation discussion

This part mainly concentrates on the validations of presented method based on simulations. In the imaging area, four targets are at 50m, 100m, 150m and 200m are focused, and the targets are labeled T1, T2, T3 and T4, individually. The corresponding azimuth positions are 5m, 8m, 12m and 15m, individually. The SAS system owns 50 receivers, and the total aperture of receiver array is 1.5 m. The aperture of emitter is 0.06 m. The carrier frequency of sonar system uses 150000 Hz. 20000 Hz is used by the chirp waveform bandwidth. The interval of sent chirp is 300 ms. Here, the sonar is operated with 2.475 m/s. Based on this speed, the system should need 49.5 receivers. Traditional nonuniform reconstruction method can well work in this case. The performance of presented method can be compared to the high performance of traditional method. Based on these configurations, we discover that the SAS system is nonuniformly sampled by the multiple receiver sensors installed along the moving direction. With this case, the forward deviation of consecutive two pulses is shorter than the real aperture of a single receiver, and there are no redundant receivers. Due to this reason, we call it the case of slight deviation.

**Fig 4:**
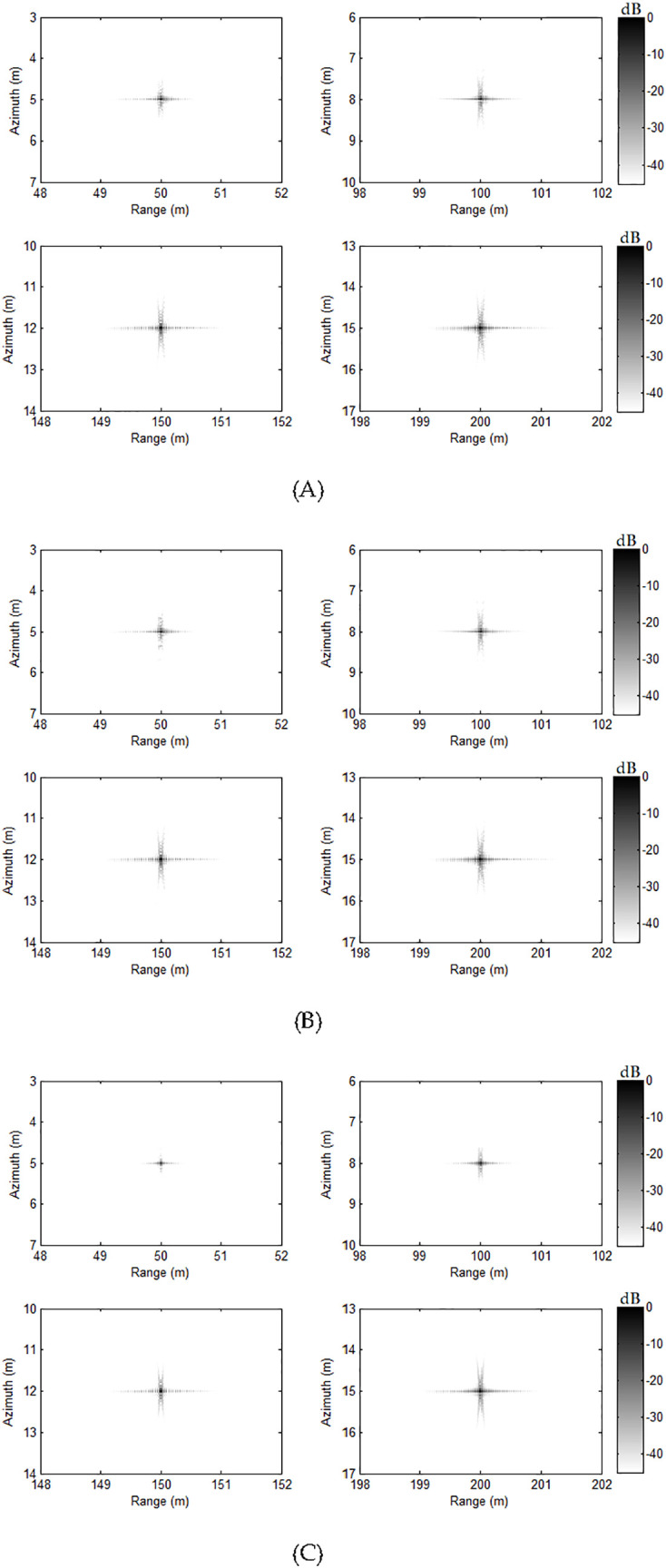
Imaging results with slight deviation. (A) FB approach, (B) our approach, (C) BP algorithm.

Using the FB method [41], presented approach in this paper and back projection (BP) algorithm [56], the handling images are acquired. Here, the images using BP algorithm are the benchmark. Fig 4A demonstrates the results using FB technique, and Fig 4B displays images via the presented approach. Fig 4C shows the results of BP method. Comparing imaging outcomes of three methods, it is apparent that three methods can precisely focus all targets. It shows that our approach in this paper can get the high-resolution image when the moving deviation between two adjacent pulses is not less than the real aperture of a single receiver.

**Fig 5:**
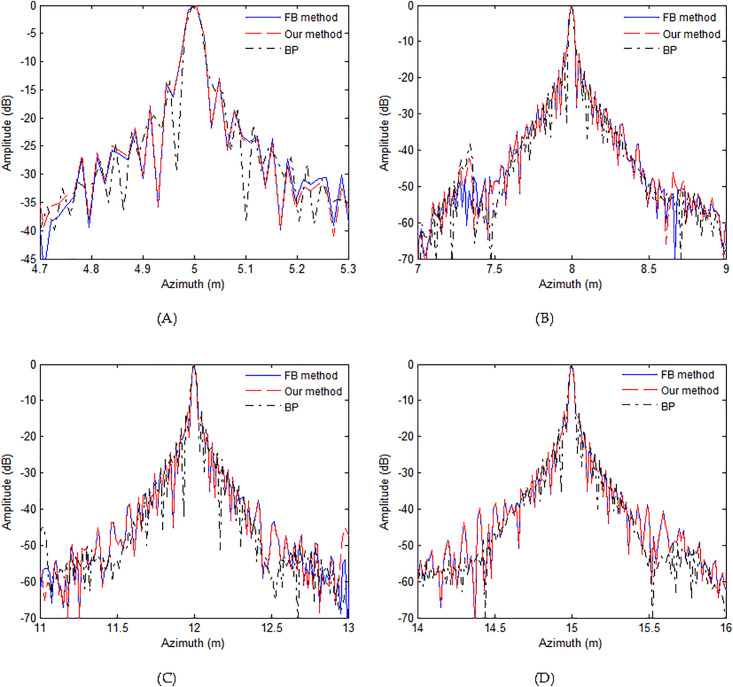
Azimuth profiles with slight deviation. (A) T1, (B) T2, (C) T3, (D) T4.

Fig 5 illustrates the azimuth profiles of four targets. As seen in the profiles of all targets, the provided approach mostly achieves identical profiles relying on the FB method [41] and BP algorithm [56]. This still proves the efficacy of provided approach.

The quality metrics can assist us to assess the performance of various approaches quantitatively. We would compute the quality metrics to further assess the quality of both methods. In Table 1, the quality metrics such as the peak sidelobe ratio (PSLR), integrated sidelobe ratio (ISLR) and azimuth resolution (AR) are adopted to compare the focusing quality of both approaches. Based on the quality metrics in Table 1, the quality metrics of presented approach is slightly inferior to those of FB method [41] and BP algorithm [56]. These distinctions are generally so insignificant that these differences can be disregarded. The discussed technique is capable of obtaining a fine image. That is to say, the presented method can get the high-resolution image. The findings gathered from Fig 4 and Fig 5 are generally in agreement with that based on Table 1.

**Table 1:** Imaging quality with slight deviation.

	FB method			Presented approach			BP		
	PSLR(dB)	ISLR(dB)	AR(m)	PSLR(dB)	ISLR(dB)	AR(m)	PSLR(dB)	ISLR(dB)	AR(m)
T1	−13.36	−10.62	0.03	−13.25	−10.48	0.03	−13.51	−10.63	0.03
T2	−13.54	−10.57	0.03	−13.49	−10.56	0.03	−13.63	−10.69	0.03
T3	−13.15	−10.49	0.03	−13.07	−10.48	0.03	−13.36	−10.71	0.03
T4	−12.97	−10.35	0.03	−12.92	−10.35	0.03	−13.27	−10.46	0.03

**Fig 6:**
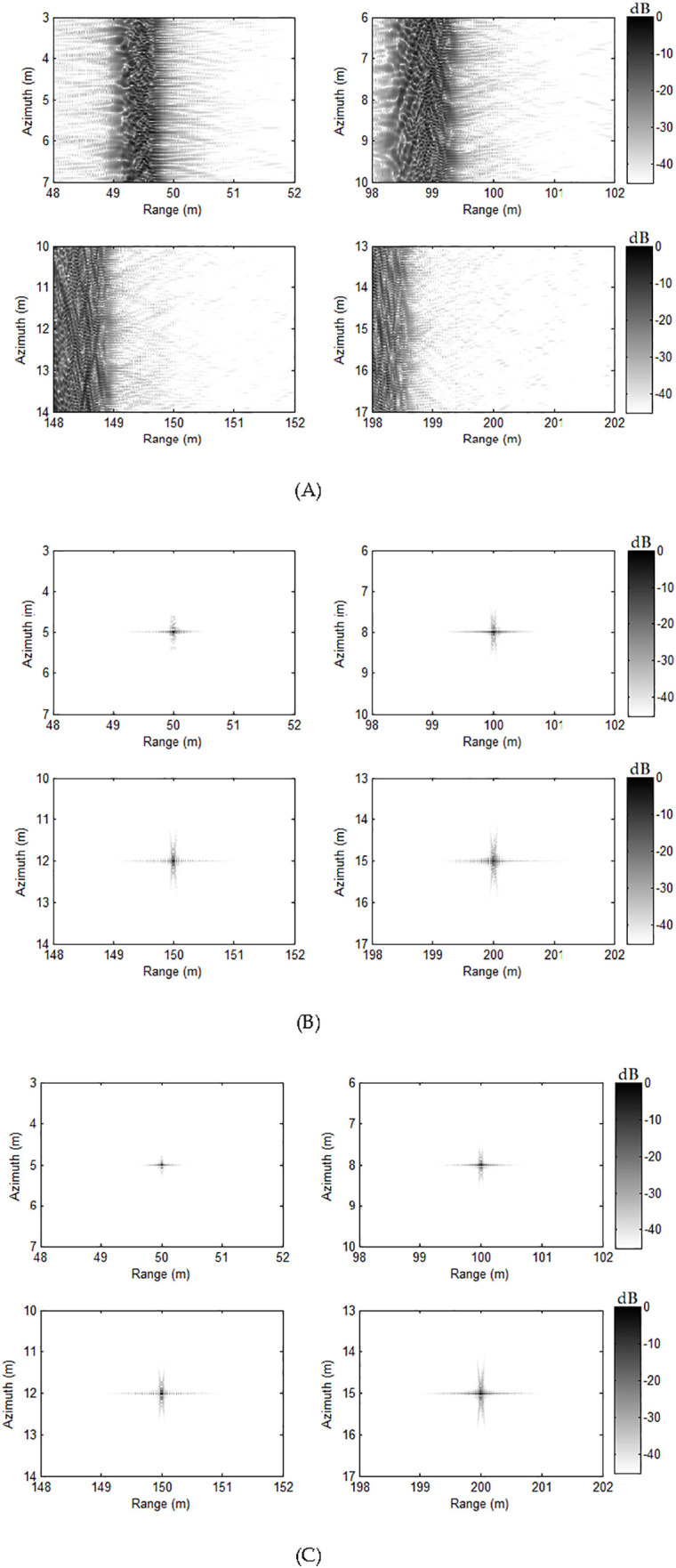
Imaging results with major deviation. (A) FB approach, (B) our approach, (C) BP algorithm.

The second simulation concentrates on the major deviation between two adjacent pulses. In this simulation, the towed speed of sonar system is changed to 2.2 m/s. Based on this speed, the system needs 44 receivers, and 6 redundant receivers are exited. With this case, traditional nonuniform reconstruction method cannot well reconstruct the signal. However, our method can still work with this case. The other parameters are identical to parameters of previous case. Based on the parameters of this case, it should be noted that there are 6 redundant receivers. Using the FB method [41], discussed method and BP algorithm [56], four targets are focused. Fig 6 exhibits the handling outcomes. The outcomes related to FB approach [41] are illustrated in Fig 6A. We can clearly see that the targets are failed to be focused. Actually, the redundant receivers make the failure of reconstruction filters, which is based on the matrix inversion. Due to this reason, the targets cannot be successfully focused. Based on the discussed approach, all targets can be still focused with the high-resolution. The performance between our method and BP algorithm is nearly the same. This shows that our approach is better than FB method [41] when the SAS system works in the case of major deviation.

**Fig 7:**
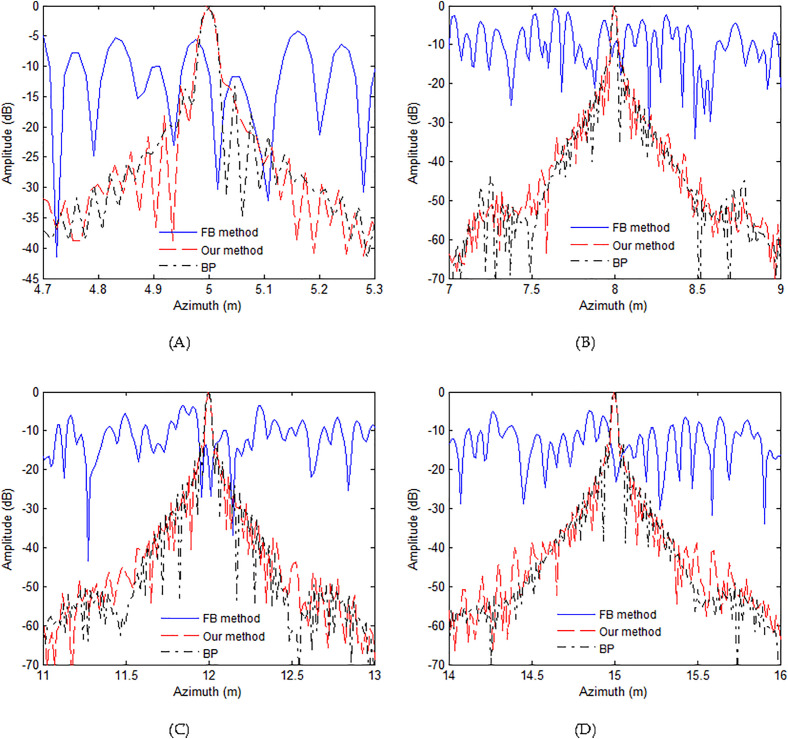
Azimuth profiles with major deviation. (A) T1, (B) T2, (C) T3, (D) T4.

Here, we still pay attention to the profiles in the azimuth dimension. The profiles related to focused targets in the azimuth dimension are presented in Fig 7. Table 2 shows the quality metrics including PSLR, ISLR and AR. Based on Fig 7 and Table 2, the FB method cannot focus the targets any more. With the presented approach, the targets are well focused. The quality parameters based on our method are slightly inferior to those of BP algorithm. In conclusion, the suggested technique is capable of significantly enhancing the handing quality even if the system operates with the major deviation case. As a result, we figure out that the suggested solution is capable of achieving outstanding outcomes in any cases.

**Table 2:** Imaging quality with major deviation.

	FB method			Presented approach			BP		
	PSLR(dB)	ISLR(dB)	AR(m)	PSLR(dB)	ISLR(dB)	AR(m)	PSLR(dB)	ISLR(dB)	AR(m)
T1	—	—	—	−12.93	−10.62	0.03	−13.12	−10.71	0.03
T2	—	—	—	−13.12	−10.64	0.03	−13.42	−10.80	0.03
T3	—	—	—	−13.11	−10.47	0.03	−13.27	−10.63	0.03
T4	—	—	—	−12.95	−10.33	0.03	−13.13	−10.51	0.03

### 4.2. Experiment and discussions

**Fig 8:**
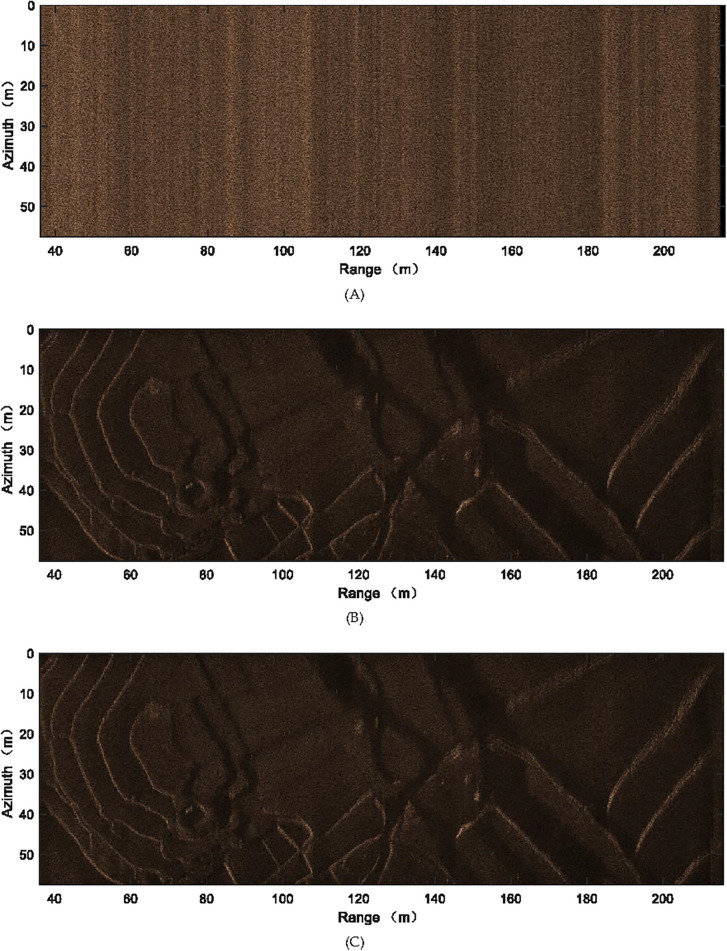
Imaging results of real SAS with major deviation. (A) FB approach, (B) Presented approach, (C) BP algorithm.

Currently, the focusing in the case of major deviation is discussed. With our system, 48 receivers are exploited and the real aperture of each receiver in the azimuth dimension is 0.04 m. The carrier frequency and bandwidth of the chirp waveform are 150000 Hz and 20000 Hz, respectively. The towed speed of sonar system is 2.5 m/s, and PRI is 0.32 s. With these parameters, it is easily found that the system suffers from 8 redundant receivers. Exploiting the FB approach [41], proposed approach and BP [56], the real datasets are handled. Fig 8A shows the outcome of FB approach. The outcome of suggested approach can be seen in Fig 8B. The outcome based on BP algorithm is presented in Fig 8C. Since discussed SAS system suffers from many redundant receivers, the reconstruction filters of FB method [41] based on the computation of matrix inversion would be not a success job. Consequently, the targets cannot be focused by exploiting the FB approach [41], and Fig 8A confirms this conclusion. The presented method well solves this issue. Therefore, the target can be successfully focused, and Fig 8B confirms this conclusion. Furthermore, the performance based on our method in Fig 8B is close to that based on BP algorithm in Fig 8C. This further verifies the efficacy of our approach. The handling time of this experiment is recorded. The FB approach and our approach need 848 s and 145 s, respectively. The BP algorithm needs 7531 s. Based on the experiment in this part, we can get the conclusion that our method is much better than FB method in the case of major deviation.

In Table 3, we still present the entropy and contrast [57] corresponding to images in Fig 8. Based on Table 3, the entropy and contrast of FB method dramatically deviate from corresponding parameters of our method and BP algorithm. That is to say, traditional method fails to recover the SAS image. Comparing the entropy and contrast of our method and those of BP algorithm, the parameter differences between our method and BP algorithm can be disregarded. It means that, our method can recover the SAS image with high quality.

**Table 3:** Imaging quality with the major deviation case.

	Entropy	Contrast
FB approach	6.53	1.48
Presented method	5.10	2.85
BP algorithm	4.96	3.02

## 5. Conclusion

This article offers the RD algorithm for SAS made up of many receiver sensors. With our approach, the receivers applied to our imaging processor are determined. Then, the 2D spectrum including the bistatic term and monostatic equivalent term is deduced based on the quadratic expansion of emitter and receiver phases. With the help of the block correction approach, tackling the bistatic term would yield the monostatic equivalent term, which is further compensated based on the quadratic decoupling, linear decoupling and azimuth compression. Using our approach, the multireceiver SAS datasets can well be focused when the SAS works in the case of slight deviation. In the case of major deviation, our method can still get the high-resolution image while FB method suffers from serious distortion. The focused results based on the simulated datasets and real SAS datasets verify the merits of our approach.

Based on our method, we can process the SAS datasets with high performance all the time in any cases. However, traditional reconstruction method just works in slight nonuniform case. Our work does not consider the motion compensation, which is our future work.

## Supporting information

S1 File This is the raw data file of experiment. (XLSX)

## References

[1] Reed A, Kim J, Blanford T, Pediredla A, Brown D, Jayasuriya S. Neural volumetric reconstruction for coherent synthetic aperture sonar. ACM Trans Graph. 2023;42(4):1–20. doi: 10.1145/3592141

[2] Zhang X, Dai X, Fang B. A range-Doppler imaging method for the multireceiver synthetic aperture sonar. Geomatics and Information Science of Wuhan University. 2019;44:1667–73.

[3] Huang P, Ding W, Wang X, Teng X. Review of interferometric synthetic aperture sonar interferometric phase filtering methods. Advances in Mechanical Engineering. 2023;15(9). doi: 10.1177/16878132231201148

[4] Wang M, Huang P. A multireceiver SAS imaging algorithm and optimization. IEEE Access. 2023;11:75112–20.

[5] Zhang X, Yang P, Sun M. Experiment results of a novel sub-bottom profiler using synthetic aperture technique. Current Science. 2022;122(4):461. doi: 10.18520/cs/v122/i4/461-464

[6] Marston TM, Bassett C, Plotnick DS, Kidwell AN, Honegger DA. Three-dimensional observations of tidal plume fronts in estuaries using a synthetic aperture sonar array. J Acoust Soc Am. 2023;154(2):1124–37. doi: 10.1121/10.0020671 37606356

[7] Marston TM, Hall BR, Bassett C, Plotnick DS, Kidwell AN. Motion tracting of fish and bubble clouds in synthetic aperture sonar data. J Acous Soc Am. 2024;155:2181–91. 10.1121/10.0025384 38512016

[8] Zhang X, Dai X, Yang B. Fast imaging algorithm for the multiple receiver synthetic aperture sonars. IET Radar Sonar & Navi. 2018;12(11):1276–84. doi: 10.1049/iet-rsn.2018.5040

[9] Huang C, Zhang H, Zhao J, Yu Y, Zhao X. Unsupervised terrain reconstruction from side-scan sonar constrained to the imaging mechanism. IEEE Trans Geosci Remote Sensing. 2024;62:1–15. doi: 10.1109/tgrs.2024.3481037

[10] Zhu J, Yin T, Guo W, Zhang B, Zhou Z. An underwater target azimuth trajectory enhancement approach in BTR. Applied Acoustics. 2025;230:110373. doi: 10.1016/j.apacoust.2024.110373

[11] Zhang X, Yang P. Imaging algorithm for multireceiver synthetic aperture sonar. J Electr Eng Technol. 2019;14(1):471–8. doi: 10.1007/s42835-018-00046-0

[12] Kiang C-W, Kiang J-F. Imaging on underwater moving targets with multistatic synthetic aperture sonar. IEEE Trans Geosci Remote Sensing. 2022;60:1–18. doi: 10.1109/tgrs.2022.3220708

[13] Zheng L, Hu T, Zhu J. Underwater sonar target detection based on improved ScEMA-YOLOv8. IEEE Geosci Remote Sens Lett. 2024;21:1503505.

[14] Zhu J, Xie Z, Jiang N, Song Y, Han S, Liu W, et al. Delay-doppler map shaping through oversampled complementary sets for high-speed target detection. Remote Sensing. 2024;16(16):2898. doi: 10.3390/rs16162898

[15] Luo Y, Liu Q, Zhang Y, Zhou H, Zhang J, Cao X. Review of underwater image object detection based on deep learning. Journal of Electrical Engineering & Technology. 2023;45:3468–82.

[16] Cheong M, Kim J, Park D-H, Kim H-N. Temporal-feature-based classification of active sonar targets in a deep-water environment. IEEE Trans Aerosp Electron Syst. 2024;60(5):7380–92. doi: 10.1109/taes.2024.3417430

[17] Liu X, Zhu H, Song W, Wang J, Yan L, Wang K. Research on Improved VGG-16 model based on transfer learning for acoustic image recognition of underwater search and rescue targets. IEEE J Sel Top Appl Earth Observations Remote Sensing. 2024;17:18112–28. doi: 10.1109/jstars.2024.3459928

[18] Malkasse J-P, Dugelay S, Burlet N, Gall YL, Simon M. Mutiple views in single-path synthetic aperture sonar for mine counter-measures classification and pre-identification. Electron Lett. 2023;59:e12822.

[19] Jiao W, Zhang J, Zhang C. Open-set recognition with long-tail sonar images. Expert Systems with Applications. 2024;249:123495. doi: 10.1016/j.eswa.2024.123495

[20] Xu Z, Xu D, Lin L, Song L, Song D, Sun Y, et al. Integrated object detection and communication for synthetic aperture radar images. IEEE J Sel Top Appl Earth Observations Remote Sensing. 2025;18:294–307. doi: 10.1109/jstars.2024.3495023

[21] Huang P, Yang P. Synthetic aperture imagery for high-resolution imaging sonar. Front Mar Sci. 2022;9:1049761.

[22] Yang P. An imaging algorithm for high-resolution imaging sonar system. Multimed Tools Appl. 2023;83(11):31957–73. doi: 10.1007/s11042-023-16757-0

[23] Zhang X, Yang P. Back projection algorithm for multi-receiver synthetic aperture sonar based on two interpolators. JMSE. 2022;10(6):718. doi: 10.3390/jmse10060718

[24] Zhang X, Yang P, Cao D. Synthetic aperture image enhancement with near-coinciding nonuniform sampling case. Computers and Electrical Engineering. 2024;120:109818.

[25] Zhang X, Yang P, Sun H. Frequency‐domain multireceiver synthetic aperture sonar imagery with Chebyshev polynomials. Electronics Letters. 2022;58(25):995–8. doi: 10.1049/ell2.12513

[26] Tan C, Zhang X, Yang P, Sun M. A novel sub-bottom profiler and signal processor. Sensors (Basel). 2019;19(22):5052. doi: 10.3390/s19225052 31752419 PMC6891379

[27] Tao X, Mengdao X, Yong W, Rui G, Jialian S, Zheng B. Using derivatives of an implicit function to obtain the stationary phase of the two-dimensional spectrum for bistatic SAR imaging. IEEE Geoscience and Remote Sensing Letters. 2011;8:1165–9.

[28] Wang H, Sun B, Li C, Jiang Y, Yang W. An improved range-doppler imaging algorithm based on high-order range model for near-field panoramic millimeter-wave ArcSAR. IEEE Trans Geosci Remote Sensing. 2023;61:1–16. doi: 10.1109/tgrs.2023.3290646

[29] Zhang X, Yang P, Dai X. Focusing multireceiver SAS data based on the fourth-order legendre expansion. Circuits Syst Signal Process. 2018;38(6):2607–29. doi: 10.1007/s00034-018-0982-6

[30] Fan N, Wang Y. Research on modified range-Doppler algorithm of synthetic aperture sonar imaging. Computer Simulation. 2014;31:25–9.

[31] Zhen Tian, Heping Zhong, Jinsong Tang, Sen Zhang. Focusing multiple receiver SAS data using extended range doppler algorithm based on series reversion and data fusion. Proceedings 2013 International Conference on Mechatronic Sciences, Electric Engineering and Computer (MEC), 2013. 3630–5. doi: 10.1109/mec.2013.6885630

[32] Zhong H, Tang J, Tian Z, Wu H, Ma M. Accelerating range Doppler imaging algorithm for multiple-receiver synthetic aperture sonar on multi-core-based architectures. Soft Computing. 2020;24:9777–88.

[33] Yang H, Tang J, Li Q, Liu X. A robust multiple-receiver Range-Doppler algorithm for synthetic aperture sonar imagery. OCEANS 2007 - Europe, Aberdeen, Scotland, UK, 2007. 1–5.

[34] Zhang X, Tang J, Zhong H, Zhang S. Wavenumber-domain imaging algorithm for wide-beam multi-receiver synthetic aperture sonar. Journal of Harbin Engineering University. 2014;35:93–101.

[35] Zhang X, Yang P, Sun H. An omega-k algorithm for multireceiver synthetic aperture sonar. Electronics Letters. 2023;59:1–3.

[36] Zhang X, Yang P. An Improved imaging algorithm for multi-receiver SAS system with wide-bandwidth signal. Remote Sensing. 2021;13(24):5008. doi: 10.3390/rs13245008

[37] Lurton X, Augustin J-M. A Measurement quality factor for swath bathymetry sounders. IEEE J Oceanic Eng. 2010;35(4):852–62. doi: 10.1109/joe.2010.2064391

[38] Kerstens R, Laurijssen D, Steckel J. An optimized planar mimo array approach to in-air synthetic aperture sonar. IEEE Sensors Letters. 2019;3:1–4.

[39] Zhang S, Tang J, Yang H. Synthetic aperture sonar image reconstruction with variational non-uniform sampling in azimuth. Chinese Journal of Sensors and Actuators. 2009;22: 230–4.

[40] Yih-Chyun Jenq. Perfect reconstruction of digital spectrum from nonuniformly sampled signals. IEEE Trans Instrum Meas. 1997;46(3):649–52. doi: 10.1109/19.585419

[41] Yang H, Tang J, Li Q, Liu X. Processing of non-uniform azimuth sampling in multiple-receiver synthetic aperture sonar image. Oceans 2007, Aberdeen, UK, 2007. 1–4.

[42] Krieger G, Gebert N, Moreira A. Unambiguous SAR signal reconstruction from non-uniform displaced phase centre sampling. IEEE Geosci Remote Sens Lett. 2004;1:260–4.

[43] Zhu Z, Zhang Z, Wang R, Guo L. Out-of-band ambiguity analysis of nonuniformly sampled SAR signals. IEEE Geoscience and Remote Sensing Letters. 2014;11:2027–31.

[44] Praczyk T. Neural control system for a swarm of autonomous underwater vehicles. Knowledge-based Systems. 2023;276:110783.

[45] Zhang X, Ying W. Influence of the element beam pattern on synthetic aperture sonar imaging. Geomatics and Information Science of Wuhan University. 2022;47:133–40.

[46] Zhang X. An efficient method for the simulation of multireceiver SAS raw signal. Multimed Tools Appl. 2024;83:37351–68.

[47] Wu Q, Xing M, Shi H, Hu X, Bao Z. Exact analytical two-dimensional spectrum for bistatic synthetic aperture radar in tandem configuration. IET Radar, Sonar and Navigation. 2011;5:349–60.

[48] Wu JJ, Yang JY, Huang YL, Liu Z, Yang HG. A new look at the point target reference spectrum for bistatic SAR. Progress in Electromagnetics Research. 2011;119:363–79.

[49] Zhang X, Yang P, Feng X, Sun H. Efficient imaging method for multireceiver SAS. IET Radar Sonar and Navigation. 2022;16:1470–83.

[50] Wang X, Zhang X, Zhu S. Upsampling based back projection imaging algorithm for multi-receiver synthetic aperture sonar. 2015 International Industrial Informatics and Computer Engineering Conference, Xi’an, China, 2015. 1610–5.

[51] Yang L, Wang T, Chen Y, Gai M, Xu H. Feature reconstruction of high resolution SAR imagery based on low rank matrix completion. Journal of Electrical Engineering & Technology. 2023;45:2965–74.

[52] Chen Q, Liu W, Sun G, Li D, Xing M. An accelerated back-projection algorithm based on large swath for geosynchronous-earth-orbit SAR imaging. Journal of Electrical Engineering & Technology. 2022;44:3136–43.

[53] Bie B, Liu J, Sun G, Wang D, Xing M. Low-orbit bistatic frequency modulated continuous wave SAR imaging method based on singular value decomposition. Journal of Electrical Engineering & Technology. 2023;45:2502–10.

[54] Zhang X, Chen X, Qu W. Influence of the stop-and-hop assumption on synthetic aperture sonar imagery. 2017 IEEE 17th International Conference on Communication Technology (ICCT), 2017. 1601–7. doi: 10.1109/icct.2017.8359901

[55] Cumming I, Wong F. Digital processing of synthetic aperture radar data: algorithms and implementation. Norwood, Ma, USA: Artech House. 2005.

[56] Lou Y, Lin H, Li N, Xing M, Wang J, Wu Z. A Prior 2-D autofocus algorithm with ground cartesian BP imaging for curved trajectory SAR. IEEE J Sel Top Appl Earth Observations Remote Sensing. 2024;17:2422–36. doi: 10.1109/jstars.2023.3346942

[57] Yang J, Li W, Li K, Chen R, Zhang K, Mao D, et al. Sparse Bayesian learning-based multichannel radar forward-looking superresolution imaging considering grid mismath. IEEE J Sel Topics Appl Earth Observ Remote Sens. 2024;17:14997–5008.

